# From intelligence to achievement: the role of the socio-cultural environment of underrepresented minority students

**DOI:** 10.3389/fpsyg.2024.1495434

**Published:** 2024-12-23

**Authors:** Olutola Opeyemi Akindipe

**Affiliations:** Department of Psychology, University of North Florida, Jacksonville, FL, United States

**Keywords:** achievement, intelligence, students, minority, sociocultural

## 1 Introduction

Minority students continue to underachieve in comparison to their majority counterparts across the country (Weinberg, 1989; Phillips, 2000). This achievement gap exists at almost all educational level and has been found to remain even when all other variables such as individual, family, and institutional factors are held constant (Brooks-Gunn et al., 1996) leading to significantly negative educational outcomes such as lower enrollment and graduation rates, high dropout rates, loss of academic opportunities, and poor career advancement among others (Huffman et al., 2003; Musu-Gillette et al., 2018). Interestingly, this underachievement has often been linked to the absence of intelligence (Hernstein and Murray, 1994; Eysenck, 1971).

## 2 Achievement and intelligence

The assessment of students' performances across the formal educational system is premised on achievement and not intelligence. Achievement is defined in specific terms as “performance outcomes that indicate the extent to which a person has accomplished specific goals that were the focus of activities in instructional environments, specifically in school, college, and university” (Steinmayr et al., 2015). In contrast, intelligence is conceived in general terms, often based on how individuals process information to solve life's problems and to adapt to the environment (Neisser et al., 1996). Achievement tests are used to assess students for placement into programs designed for the highly intelligent, such as the gifted programs, while the assessment of intelligence constructs like goals setting, problem solving, information processing, creativity, novelty, critical thinking, and self-monitoring are seldom conducted (Kaplan, 2019). Although intelligence is a main predictor of academic success and should naturally translate into high achievement, it is usually not the case (Rhode and Thompson, 2007; Deary et al., 2007); more so for underrepresented minority students facing several personal, family, and institutional debilitating factors.

## 3 The sociocultural context and intelligence

Acknowledging the intelligence of underrepresented minority students and situating their learning accordingly is critical to their academic achievement. One viewpoint of intelligence alludes that it is genetically exclusive to white people and that the minority population such as the Hispanic people are not intelligent (Hernstein and Murray, 1994; Eysenck, 1971). Another perspective opines that intelligence is socially constructed and all humans, irrespective of ethnicity, are intelligent. Several intelligence theories have also given credence to the latter stance. For example, Sternberg's (1985) triarchic theory of intelligence proposed analytical, creative, and practical intelligence as products of individual's social experiences which aid problem solving and adaptation to the environment. Also, the socio-cultural (Vygotsky, 1978) and the situated cognition theories of intelligence (Brown et al., 1989) focus on the role of individuals' socio-cultural experiences in the development of cognition and intelligence.

In addition, Bernstein's (1966) code theory postulated that the structures of social classes create two language codes, restricted and elaborated, which influence intelligence. He stated that the working- and middle-class families could use the restricted code which relied on implicit meaning, simple vocabulary and sentences, however, only the middle-class families had access to the elaborated code which had structured vocabulary, complex sentences, and explicit communication. The theory, therefore, alludes that students from the middle class are more intelligent and perform academically better than working-class students because the latter cannot switch to the elaborated code used in schools, and this seemingly explains the achievement gap.

Research studies from both western and non-western cultures also provide evidence to support the importance of the socio-cultural context on intelligence. Cole et al.'s (1971) study of the Kpelle tribe, a rural community in West Africa, revealed the importance of people's social experiences on intelligence. Some of the villagers in the study, though unlearned, demonstrated sophisticated strategies for sorting and categorizing objects that were not only cognitively advanced but also functional and adaptive to their unique experiences. Similarly, Carraher et al. (1985) studied a group of young and uneducated street hawkers in Brazil. The researchers discovered the natives had developed innovative problem-solving ability and abstract thinking that helped in calculating the prices of goods and the monetary change to give customers even though they had no formal education. Also, Labov (1968), in his criticism of Bernstein's code theory, demonstrated through his work with the working-class that minority children who spoke Vernacular English and raised with the restricted code could easily make propositions and logical arguments.

Understanding the role of the socio-cultural environment is pertinent to translating intelligence into high achievement for several reasons. First, the social context provides the platform for any form of formal education. Concrete examples for the development of higher scientific and abstract thinking originate from the environment, as corroborated by Piaget's (1952) cognitive theory. Also, the “zone of proximal development,” a higher cognitive structure and ability is acquired within the social environment (Vygotsky, 1978). Second, the social environment fosters the development of automatization, novelty, and creativity by creating the appropriate experiences for individuals to apply information and make connections to activate new perspectives of thinking and intelligence (Sternberg, 1985). Third, social interactions provide the tool for language development which influences our thinking, cognition, and intelligence. Because we cannot think or reason beyond what we have language for, society invariably defines and limits our intelligence through language. Concepts like “self-talk,” the speech which emerges in children during play and used for problem solving, showcases the importance of the social environment in the development of intelligence (Vygotsky, 1978). Fourth, the structure of the social class, including the social interactions and shared experiences, influences our linguistic expression and communication which lays the foundation for higher cognitive and intellectual abilities (Bernstein, 1966).

Failure to acknowledge and incorporate the sociocultural environment of minority students into their education will result in them being labeled as “unintelligent” and a greater achievement gap. When schoolteachers and officials recognize students' unique cultural background, they will see the students as different rather inferior, resulting in the willingness to nurture them academically. For example, such teachers will not only believe that the restricted code is simply different but will be more inclined to help them learn the elaborated code that they need to academically succeed. Also, ignoring the students' sociocultural context would lead to loss of identity, lack of motivation to learn, poor psychological wellbeing, low self-esteem, and identity confusion (Sandhu et al., 2012; Nasir et al., 2009). Relatedly, students would lose trust and confidence in the educational system which will engender psychological alienation and dissociation and further result in academic failure, academic disempowerment, and in extreme cases, dropping out (Morris and Monroe, 2009; Saddler, 2005). In addition, students could experience stereotype threat, a form of anxiety that emanates from the perception that they will be negatively evaluated in accordance with the stereotype that minority students are not intelligent (Steele and Aronson, 1995). Consequently, this fear would inhibit the efficiency of their working memory to cognitively process information and solve problems during testing, thereby plunging them into further underperformance.

## 4 Conclusion

While acknowledging that there are several factors that impinge upon minority students' academic achievement and interventions that can be implemented at the district, state, and national levels, failure to incorporate the unique sociocultural experiences of underrepresented minority students into their education may lead to an overlook of their intelligence and further widening of the achievement gap.

## References

[B1] Bernstein B. (1966). “Elaborated and Restricted Codes: Their Social Origins and some Consequences,” in Communication and Culture, ed. A. G. Smith (Evanston: Holt Rinehart and Winston).

[B2] Brooks-Gunn J. Klebanov P. Duncan G. (1996). Ethnic differences in children's intelligence 101 test scores: role of economic deprivation, home environment, and maternal characteristics. Child Dev. 67, 396–408. 10.2307/1131822 8625720

[B3] Brown J. S. Collins A. Duguid P. (1989). Situated cognition and the culture of learning. Educ. Res. 18, 32–42. 10.3102/0013189X01800103238293548

[B4] Carraher T. N. Carraher D. W. Schliemann A. D. (1985). Mathematics in the streets and in schools. Br. J. Dev. Psychol. 3, 21–29. 10.1111/j.2044-835X.1985.tb00951.x

[B5] Cole M. Gay J. Glick J. A. Sharp D. W. (1971). The Cultural Context of Learning and Thinking: An Exploration in Experimental Anthropology. New York: Basic Books, Inc., Publishers.

[B6] Deary I. J. Strand S. Smith P. Fernandes C. (2007). Intelligence and educational achievement. Intelligence 35, 13–21. 10.1016/j.intell.2006.02.001

[B7] Eysenck H. J. (1971). Race, intelligence and education. New Soc. 17, 1045–1047.

[B8] Hernstein R. J. Murray C. A. (1994). The Bell Curve: Intelligence and Class Structure in American Life. New York: Free Press.

[B9] Huffman K. Liagas C. Snyder T. D. (2003). Status and Trends in the Education of Blacks. ED Pubs.: National Center for Educational Statistics.

[B10] Kaplan D. (2019). Creativity in education: teaching for creativity development. Psychology 10, 140–147. 10.4236/psych.2019.102012

[B11] Labov W. (1968). “The logic of non-standard English,” in Languages and Linguistics, ed. J. Alatis (Washington: Georgetown Monograph), 1–31.

[B12] Morris J. E. Monroe C. R. (2009). Why study the U.S. south? The nexus of race and place in investigating black student achievement. Educ. Res. 38, 21–36. 10.3102/0013189X0832887638293548

[B13] Musu-Gillette L. Zhang A. Wang K. Zhang J. Kemp J. Diliberti M.. (2018). Indicators of School Crime and Safety: 2017 (NCES 2018-036/NCJ 251413). Washington, DC: National Center for Education Statistics, U.S. Department of Education and Bureau of Justice Statistics, Office of Justice Programs, U.S. Department of Justice.

[B14] Nasir N. S. McLaughlin M. W. Jones A. (2009). What does it mean to be African American? Constructions of race and academic identity in an urban public high school. Am. Educ. Res. J. 46, 73–114. 10.3102/000283120832327938293548

[B15] Neisser U. Boodoo G. Bouchard T. J. Jr. Boykin A. W. Brody N. Ceci S. J.. (1996). Intelligence: knowns and unknowns. Am. Psychol. 51, 77–101. 10.1037/0003-066X.51.2.77

[B16] Phillips M. (2000). “Understanding ethnic differences in academic achievement: empirical lessons from national data,” in Analytic Issues in the Assessment of Student Achievement, eds D. W. Grissmer, and J. M. Ross (Washington, DC: U.S. Department of Education. National Center for Education Statistics), 103–132.

[B17] Piaget J. (1952). The Origins of Intelligence in Children. New York: International Universities Press. 10.1037/11494-000

[B18] Rhode T. E. Thompson L. A. (2007). Predicting academic achievement with cognitive ability. Intelligence 35, 83–92. 10.1016/j.intell.2006.05.004

[B19] Saddler C. A. (2005). The impact of brown on African American students: a critical race theoretical perspective. Educ. Stud. 37, 41–55. 10.1207/s15326993es3701_5

[B20] Sandhu D. Singh B. Tung S. Kundra N. (2012). Adolescent identity formation, psychological well-being, and parental attitudes. Pakistan J. Psychol. Res. 27, 89–105. Available at: https://link.gale.com/apps/doc/A317903015/AONE?u=jack91990&sid=bookmark-AONE&xid=55e44be4

[B21] Steele C. M. Aronson J. (1995). Stereotype threat and the intellectual test performance of African Americans. J. Person. Soc. Psychol. 69, 797–811. 10.1037/0022-3514.69.5.797 7473032

[B22] Steinmayr R. Meibner A. Weidinger A. Wirthwein L. (2015). Academic Achievement. Available at: 10.1093/obo/9780199756810-0108 (accessed November 24, 2023).

[B23] Sternberg R. J. (1985). Beyond IQ: A Triarchic Theory of Intelligence. Cambridge: Cambridge University Press.

[B24] Vygotsky L. S. (1978). Mind in Society. Cambridge, MA: Harvard University.

[B25] Weinberg R. (1989). Intelligence and IQ: landmark issues and great debates. Am. Psychol. 44, 98–104. 10.1037/0003-066X.44.2.98

